# In Brief: Kanavel’s Signs and Pyogenic Flexor Tenosynovitis

**DOI:** 10.1007/s11999-015-4367-x

**Published:** 2015-05-29

**Authors:** Colin D. Kennedy, Jerry I. Huang, Douglas P. Hanel

**Affiliations:** Department of Orthopaedics and Sports Medicine, University of Washington, 1959 NE Pacific Street, Box 356500, Seattle, WA 98195-6500 USA

## History

Pyogenic flexor tenosynovitis is an infection of the flexor tendon sheath of the finger that can result in tendon necrosis and adhesions leading to marked loss of motion, deformity, and loss of limb, particularly if treatment is delayed [21, 22]. In one large series, pyogenic flexor tenosynovitis was reported to represent 9.4% (13/138) of hand infections [7]. The advent of antibiotics and appropriate surgical treatment has decreased the risk of serious sequelae secondary to pyogenic flexor tenosynovitis. However, early recognition and clinical suspicion remain paramount to minimizing potentially devastating consequences from delayed treatment of these infections.

Dr Allen B. Kanavel (1874–1938) initially described three cardinal signs of pyogenic flexor tenosynovitis in his seminal work in 1912 as “1. Exquisite tenderness over the course of the sheath, limited to the sheath. 2. Flexion of the finger. 3. Exquisite pain on extending the finger, most marked at the proximal end” [10]. Although not noted in his initial description as a cardinal sign, he explained “the whole of the involved finger is uniformally swollen,” and fusiform swelling later became the fourth cardinal sign [10, 11]. The constellation of the four signs, commonly known as “Kanavel’s signs,” is frequently used as the primary clinical tool for diagnosing pyogenic flexor tenosynovitis, because advanced imaging and laboratory studies often are nonspecific [4].

## Purpose

Understanding the common presentations of pyogenic flexor tenosynovitis allows for timely diagnosis of the condition to allow prompt treatment to take place. A useful clinical tool also would help the clinician exclude the diagnosis of pyogenic flexor tenosynovitis when it is not present to allow correct identification of other kinds of disorders that should be included in the initial differential diagnosis for a swollen or painful finger. Conditions that mimic acute pyogenic flexor tenosynovitis include abscesses, felons, herpetic whitlow (a cutaneous infection caused by the herpes simplex virus often presenting in medical and dental professionals with clear, painful vesicles that coalesce in painful bullae over the fingertip), gouty arthritis, and septic arthritis involving the metacarpophalangeal or interphalangeal joints [4, 22].

## Description

**Fig. 1:**
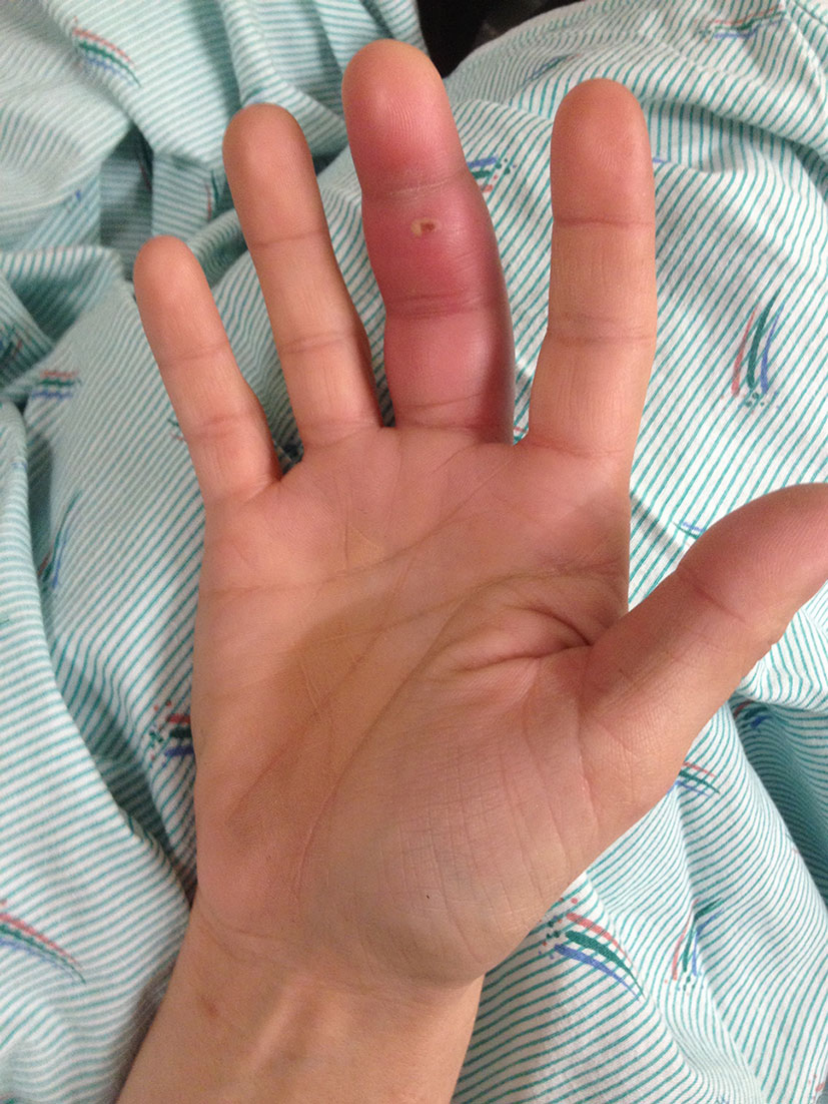
The right middle finger of this patient with pyogenic flexor tenosynovitis shows fusiform swelling and the digit was held in flexion with tenderness to palpation along the flexor tendon sheath and exquisite pain with passive digit extension. There is an identifiable entry site of previous trauma overlying the middle phalanx. (Published with permission from Alexander Lauder MD, Department of Orthopaedics and Sports Medicine, University of Washington, Seattle, WA, USA.)

Pyogenic flexor tenosynovitis usually is caused by penetrating trauma to the finger, although patients who are immunocompromised may have a more indolent and chronic presentation, and a history of trauma in these patients may be remote or absent. The site of penetrating trauma often can be identified and may appear relatively mild, even appearing like nothing more than a superficial scratch (Fig. 1). Multiple series have shown *Staphylococcus aureus* as the most common pathogen in pyogenic flexor tenosynovitis cultures, although polymicrobial infections, methicillin-resistant *S aureus* (MRSA), *Staphylococcus epidermidis, Pseudomonas aeruginosa,* and *Streptococcus* species also are commonly encountered pathogens [1, 4, 5, 20], and rare organisms have been isolated from pyogenic flexor tenosynovitis cultures, including *Eikenella corrodens, Pasteurella multocida, Kingella kingae,Listeria monocytogenes, Neisseria gonorrhoeae, Clostridium difficile,* and *Mycobacterium* species [2, 9, 15, 19, 22, 23]. Initial treatment of pyogenic flexor tenosynovitis includes timely administration of intravenous antibiotics and surgical irrigation and drainage. Although open irrigation and débridement, closed tendon sheath irrigation, local antibiotic delivery systems, and continuous closed irrigation strategies using external pump local anesthesia have been described, no single approach is clearly superior to others regarding long-term function, need for repeat surgery, amputation risk, and infection eradication [1, 5, 6, 8, 16].

**Fig. 2:**
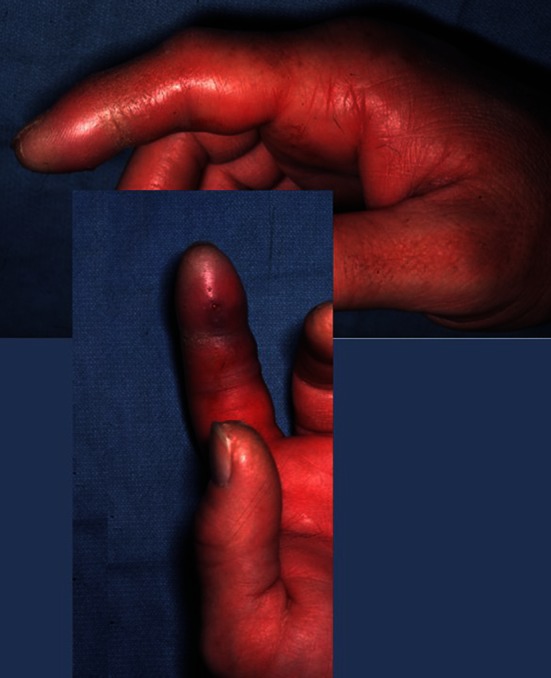
The index finger of this patient with pyogenic flexor tenosynovitis shows fusiform swelling of the digit and the digit is held in flexion. This patient had pain with passive extension of the digit and tenderness to palpation along the length of the flexor tendon sheath.

Although the absence of one or more Kanavel’s signs does not exclude a diagnosis of pyogenic flexor tenosynovitis, the classic description of exquisite tenderness along the flexor tendon sheath, the digit held in flexion at rest, fusiform swelling of the digit (often described as a “sausage digit”), and pain with passive extension of the digit should raise concern for the presence of pyogenic flexor tenosynovitis (Fig. 2).

## Validation

Using a PubMed search, there are no available published studies that have validated the sensitivity or specificity of Kanavel’s four cardinal signs for the diagnosis of pyogenic flexor tenosynovitis. Additionally, there are no studies addressing the interobserver reliability for Kanavel’s signs as a diagnostic tool for pyogenic flexor tenosynovitis.

However, several studies have evaluated the use of the sign as a diagnostic tool in other ways. Pang et al. [18] analyzed 75 patients with pyogenic flexor tenosynovitis and found that of the Kanavel’s signs, fusiform swelling was the most commonly found sign and was present in 97% (73/75) of patients. This was followed by pain on passive extension in 72% (54/75) of patients, semiflexed posture in 69% (52/75), and tenderness along the flexor sheath in 64% (48/75). They found that tenderness along the tendon sheath was a late sign of proximal extension, suggesting that the lack of this Kanavel’s sign should not exclude a diagnosis of pyogenic flexor tenosynovitis. Kanavel described excessive tenderness along the tendon sheath as the most important sign [10]. Kanavel’s signs in the small finger and thumb may be more subtle than the central fingers because these fingers have an autodecompression mechanism through the ulnar and radial bursae [22]. Neviaser and Gunther [17] found that the inability to flex the finger to touch the palm was an additional sign of pyogenic flexor tenosynovitis and suggested that the most reliable early Kanavel’s sign is pain on passive extension of the digit. Dailiana et al. [3] performed a retrospective review of 41 patients with pyogenic flexor tenosynovitis and found that only 54% (22/41) of patients had all four signs. They noted that all patients in their series had tenderness along the tendon sheath and pain with passive extension. Thus, there is no general accord regarding which Kanavel’s cardinal sign is most predictive of pyogenic flexor tenosynovitis. Additionally, no published study to date has evaluated the combined predictive probability of each of the four Kanavel’s signs, such has been seen with the widely used “Kocher criteria” for pediatric septic hip arthritis [13].

Pang et al. [18] devised a three-tier classification system of pyogenic flexor tenosynovitis based on preoperative clinical assessment that they thought might be used to guide treatment (Group I was limited to various Kanavel’s signs but no subcutaneous purulence or digital ischemia. Patients in Group II had subcutaneuous purulence, and patients in Group III had digital ischemia.) . All three tiers in their new classification showed various Kanavel’s signs. They found a significant association between increasing risk of digit amputation with higher classification tier and an inverse correlation between increasing classification group and return of total active motion. In addition, they found that age older than 43 years, poorly controlled diabetes mellitus, peripheral vascular disease, renal failure, and involvement of more than one bacterial species significantly increased the likelihood of amputation and decreased likelihood for recovery of total active motion.

## Limitations

The diagnosis of pyogenic flexor tenosynovitis can be particularly challenging in children, because pediatric patients with pyogenic flexor tenosynovitis may not show the classic Kanavel’s signs. To our knowledge, only one study has evaluated children with pyogenic flexor tenosynovitis and described three pediatric patients with operatively confirmed pyogenic flexor tenosynovitis in which two patients had all four Kanavel’s signs and the third patient did not clearly show any of the four Kanavel’s signs at initial presentation [14]. Given the long-term sequelae and morbidity of untreated pyogenic flexor tenosynovitis, one must be vigilant in suspicion for pyogenic flexor tenosynovitis in pediatric hand infections and not dismiss it as a diagnosis solely because one or more of the classic Kanavel’s signs are lacking [14].

Kanavel’s four cardinal signs frequently are used as the primary diagnostic clinical criteria for pyogenic flexor tenosynovitis, and the presence of these signs is often the main criterion used in the decision toward proceeding with operative treatment of these infections. Although it was suggested that some of the signs are present in a majority of patients [18], much less is known about the specificity of predictive value of these signs, and no study to our knowledge has shown the interobserver validity of Kanavel’s signs. The additive predictive value with Kanavel’s signs has not been shown as it has in other conditions with classic diagnostic criteria such as the “Kocher criteria” used in the diagnosis of pediatric septic hip arthritis [13]. Therefore, further data regarding which of the four Kanavel’s signs or which combination of the four signs is most strongly indicative of a pyogenic flexor tenosynovitis diagnosis would be beneficial. Retrospective data on hand infections that had positive Kanavel’s signs that were not subsequently diagnosed intraoperatively as pyogenic flexor tenosynovitis also would be beneficial to gain understanding of the specificity of the cardinal signs. Although likely difficult to formally study, the interobserver reliability of Kanavel’s signs has not been evaluated, and such data may be the most useful tested among emergency department physicians who often are the first-line providers encountering hand infections before hand surgery consultation. Further understanding of the validated sensitivity, specificity, and reliability of Kanavel’s signs for pyogenic flexor tenosynovitis diagnosis could lead to the addition to or refinement of the classic Kanavel’s signs and aid in the diagnosis of an ailment that often is misdiagnosed and can be difficult to ascertain in pediatric or immunocompromised populations.

Determining the sensitivity, specificity, and interobserver reliability presents challenges that likely explain why this has not been explicitly shown in previous publications. Pyogenic flexor tenosynovitis does not have a gold standard for diagnosis, and if one assigns purulence encountered intraoperatively as the gold standard for a diagnosis, the true sensitivity and specificity would not be readily attainable because not all patients who are examined with respect to Kanavel’s signs have surgical débridement that could be used to confirm or exclude the diagnosis and generate accurate sensitivity and specificity data. Calculating the intraobserver reliability is not feasible, and establishment of the interobserver variability testing of this rare condition requires simultaneous observation by multiple observers that usually is not practical in a rare condition that even in high volume tertiary trauma centers is not routinely encountered. Furthermore, pyogenic flexor tenosynovitis encompasses a minority of hand infections seen, even in busy Level 1 trauma centers, and obtaining enough data to generate meaningful statistical conclusions likely requires a long period of data collection or a multicenter trial.

## Conclusions/Uses

Pyogenic flexor tenosynovitis is an infection of the flexor tendon sheath in which clinical diagnosis is made using the four cardinal Kanavel’s signs. Despite appropriate antibiotics and surgical treatment, pyogenic flexor tenosynovitis can be devastating. These infections often are misdiagnosed, and delayed diagnosis is associated with worsened ROM owing to adhesions, tendon necrosis and rupture, deformity, and risk of loss of limb [3, 12, 18, 21]. In addition, other hand ailments including septic arthritis, crystal-induced arthritides, and stenosing flexor tenosynovitis can have similar presentations to pyogenic flexor tenosynovitis [4, 22]. Kanavel’s signs are a useful clinical tool for a diagnosis that otherwise lacks laboratory or radiologic signs that meaningfully contribute to accurate diagnosis. However, to date, no other clinical examination tool has proven to be superior. Given the potential morbidity of a missed or delayed diagnosis of pyogenic flexor tenosynovitis and that this infection can be present without all four Kanavel’s signs seen on initial presentation, providers should proceed with caution when using the absence of one or more Kanavel’s signs to exclude a diagnosis of pyogenic flexor tenosynovitis. Future studies might evaluate the sensitivity and specificity of Kanavel’s signs, as this could aid in preventing the incorrect diagnosis of pyogenic flexor tenosynovitis leading to overtreatment in addition to avoiding delayed or misdiagnosed pyogenic flexor tenosynovitis. Such a study requires a relatively high-volume trauma center that sees a large number of patients with hand infections and could be done by documenting which of the four Kanavel’s signs were present at initial evaluation, then retrospectively reviewing operative data regarding whether purulence and true pyogenic flexor tenosynovitis were encountered intraoperatively. These data then could be analyzed to gain understanding of not only the sensitivity and specificity of Kanavel’s signs, but also which sign or combination of signs is most predictive of pyogenic flexor tenosynovitis. Such a study is challenging as there is no clear gold standard for a diagnosis of pyogenic flexor tenosynovitis, and even retrospectively confirming the diagnosis can be difficult such as in cases with culture-negative microbiology results or in which intraoperative purulence is not encountered in the tendon sheath.
